# Oil Characterization and Lipids Class Composition of Pomegranate Seeds

**DOI:** 10.1155/2017/2037341

**Published:** 2017-07-24

**Authors:** Zahra Amri, Houda Lazreg-Aref, Manel Mekni, Sinda El-Gharbi, Olfa Dabbaghi, Beligh Mechri, Mohamed Hammami

**Affiliations:** Biochemistry Laboratory, LR12ES05 “Nutrition-Functional Foods and Vascular Health”, Faculty of Medicine, University of Monastir, Monastir, Tunisia

## Abstract

This study aims to investigate the physicochemical characteristics, phenolics content, and oil composition of pomegranate oil seeds (PSO). Quality indices, pigments, phenolics content, and antioxidant activity were determined. PSO was fractioned into polar lipids: glycolipids (GL) and phospholipids (PL). Sterols profile and fatty acids composition of total lipids (TL), GL, and PL were determined by GC/FID. The free acidity, the peroxide value, and the specific extinction coefficients were, respectively, 1.69%, 3.42 in milliequivalents of active oxygen per kilogram of oil, 4.15, and 3.95. PSO is rich in phenols (93.42 mg/Kg) but poor in pigments. The sterols markers were *β*-sitosterol (77.94%), Δ^5^-avenasterol (7.45%), and campesterol (6.35%). Oil content was 12.2%, wherein 23.9% were GL and 24.35% were PL. TL were rich in unsaturated fatty acids (63.17%), while saturated fatty acids were more present in PL and GL (71.97% and 66.29%, resp.). Conjugated fatty acids were about 13.30%, 2.03%, and 4.91%, respectively, in TL, PL, and GL. The* cis*/*trans* ratio of TL, PL, and GL was, respectively, 49.82%, 42.91%, and 27.39%. Monounsaturated fatty acids were more bound in PL, whereas polyunsaturated fatty acids were more bound in GL. PSO is a good source of essential fatty acids, phenolics compounds, phytosterols, and lipid-soluble fractions.

## 1. Introduction

Several studies have reported that consumed oils have enormous effects on human physiology, including lipid metabolism, development of chronic disease, and well-being [1]. No oil from a single source has been found to be suitable for all purposes because oils from different sources generally differ in their composition [2]. So interest in new sources of edible oils has recently grown. In this regard, plant seeds are known to be a good source of oils of nutritional, industrial, and pharmaceutical importance. Although conventional edible oils such as soybean, corn, and canola have their own importance, there are more rare and unfamiliar oils having unique characteristics and health-promoting traits. Pomegranate seeds oil (PSO) is such oil. It is considered a powerful health-benefiting agent due to its antioxidative, anticarcinogenic, and antilipidemic properties [3–5]. The composition of the fatty acids of PSO has been reported [1, 6–9], while little is known about the oil constitution, specially its minor compounds such as phenols and polar lipids. In addition, natural fats and oils contain, apart from glycerides, a number of lipophilic materials. Among the most interesting are the glycolipids, phospholipids, sterols, fat-soluble vitamins, and phenols. So the study of PSO for its minor constituents, however, can be useful in order to use both oil and the minor constituents effectively. For example, phenolic compounds have been reported to be present in all vegetable oils as secondary metabolites and they are important for the oxidative stability of the PUFA of these oils [10]. Furthermore, commercial antioxidants such as butylated hydroxyanisole (BHA), butylated hydroxytoluene (BHT), and tert-butylhydroquinone (TBHQ) [11] were usually added to food in many manufacturers to prevent quality deterioration and to maintain the nutritional value of different food products including oils and products containing oil [12]. In this work, physicochemical properties, phenolic content, pigment content, sterols composition, and fatty acids profile of PSO and its lipid classes have been analyzed. The results will be important as an indication of the potential economical utility of PSO as a new source of edible oils. Besides, to our knowledge, no study about phenolic content and lipid classes of PSO has been carried out previously.

## 2. Materials and Methods

### 2.1. Plant Materiel

Fruits sample were collected at full maturity from pomegranate trees of* Tounsi* variety in governorate of Mahdia, Tunisia, in October 2015. The grains were manually separated from the pulp, carefully washed, and dried in the sun until constant weight. Then, the grains were crushed and sieved to obtain fine powders.

### 2.2. Oil Extraction

Oil was extracted by the method of Soxhlet as described previously by Nasri and Triki (2004) [13]. About 30 g seeds were extracted with 200 ml of hexane at room temperature for 6 h. The solvent was removed by evaporation at 40°C and the oil was flushed with nitrogen stream and stored at −20°C in sealed tubes.

### 2.3. Determination of Quality Indices

Free acidity, peroxide value, and *K*270 and *K*232 were determined following the analytical methods described by Regulation EEC/2568/91 of the Commission of the European Union [14].

#### 2.3.1. Free Acidity

Free acidity was determined by titration of a solution of oil dissolved in ethanol/ether (1 : 1, vol/vol) with ethanolic solution of potassium hydroxide (0.1 M). The result was expressed as % of oleic acid.

#### 2.3.2. Peroxide Value

Peroxide value was determined by incubating a mixture of oil and chloroform/acetic acid (10 : 15, vol/vol) with a solution of potassium iodide in the dark for 5 min. Then, 25 ml of water and 500 *μ*l of Amidon 1% were added and the liberated iodine was titrated with sodium thiosulfate Na_2_S_2_O_3_ (0.01 N). Result was expressed in milliequivalents of active oxygen per kilogram of oil (meqO_2_/kg),

#### 2.3.3. Extinction Coefficients

*K*270 and *K*232 extinction coefficients were calculated by measuring absorbance at 270 and 232 nm, respectively, using a 1% solution of oil in cyclohexane and a path length of 1 cm.

### 2.4. Pigments Content

#### 2.4.1. Chlorophyll Content

The total chlorophyll content was calculated according to method of Kiritsakis (1998) [15]. Absorbance was measured at 630, 670, and 710 nm and carbon tetrachloride was used as blank. The calculation of the total chlorophyll content is as follows:

(1) $$\begin{array}{c} \text{Chlorophyll }\left(\;mg/kg\;\right)=\frac{\left[A_{670}-\left(A_{630}+A_{710}\right)/2\right]}{\left(0.901*L\right)}, \end{array}$$

where *A* is the absorbance of the oil at the respective wavelength and *L* is the cell thickness (cm).

#### 2.4.2. Beta-Carotene Content

Beta-carotene was measured according to the method described by Dhibi et al. (2014) [16] and the content was expressed using the following equation:

(2) $$\begin{array}{c} Beta\text{-}Carotene=A_{max}\times \left(\;\frac{10^{5}}{2.650}\;\right), \end{array}$$

where *A*_max_ is maximum of absorption between 440 and 480 nm

### 2.5. Phenolic Compounds Determination

#### 2.5.1. Extraction of Phenolic Fraction

Phenolic fraction was extracted following the procedure of Mraicha et al. (2010) [17] with some modifications. 4 g of oil was mixed with 2 ml of hexane and 4 ml of methanol/water (60 : 40, v/v). The mixture was shacked vigorously and centrifuged for 3 min at 1490 ×g. phenolic fraction was recuperated in the hydroalcoholic phase and the hexanic phase was reextracted twice with 4 ml of methanol/water (60 : 40, v/v) solution each time. Finally, the hydroalcoholic fractions obtained were combined, washed with 4 ml of n-hexane, and stored at −20°C.

#### 2.5.2. Colorimetric Determination of Total Phenols and O-Diphenols

Total phenols and O-diphenols were measured following method of Montedoro et al. (1992) [18] with minor modifications. For total phenols, 0.4 ml of the combined fractions was mixed with 10 ml of Folin-Ciocalteu reagent (1/10). After 1 min of incubation, 8 ml of sodium carbonate solution (75 g/l) was added and the mixture was incubated for 2 h in dark. Then absorbance was measured at 725 nm and the content was expressed as milligrams of gallic acid equivalents per kg of oil.

For O-diphenols content, 100 *μ*l of combined fractions was mixed with 1 ml of HCL solution (0.5 N), 1 ml of solution of a mixture of NaNO_2_ (10 g) and MaMoO_4_·2H_2_O (10 g) in 100 ml H_2_O and finally 1 ml of NaOH solution (1 N). After 30 min of incubation, the content of O-diphenols was measured at 500 nm and expressed as milligrams of gallic acid equivalents per kg of oil.

#### 2.5.3. Determination of Flavonoids Content

Total flavonoid content were determined used method of Bouaziz et al. (2010) [19]. One ml of extract or standard solutions of catechin was mixed with 4 ml of distiller water. Then 0.3 ml of NaNO_2_ (5%, w/v) was added. After 5 min, 0.3 ml of AlCl_3_ (10% w/v) was added and 2 ml of NaOH solution (1 M) was added after 6 min. Finally, 2.4 ml of distilled water was added to adjust final volume to 10 ml. After vigorous shaking, the absorbance was read at 510 nm. Content of flavonoids was expressed as mg catechin equivalents (CEQ)/g of sample.

### 2.6. DPPH Free Radical Scavenging Activity

The capacity of PSO to scavenge the free radical 2,2-diphenyl-1-picrylhydrazyl (DPPH) was measured according to the method described by Bouaziz et al. (2005) [20]. 0.25 ml of phenolic fraction of PSO was mixed with 0.5 ml of methanolic solution containing DPPH radicals (6 × 10^-6 ^ M). The mixture was shaken vigorously and incubated for 30 min in the dark at room temperature and absorbance was measured at 517 nm. DPPH scavenging effect was calculated as the percentage of DPPH discoloration using the following equation:

(3) $$\begin{array}{c} \%\text{ scavenging effect}=\left[\;\frac{\left(A-AE\right)}{A}\;\right]\times 100, \end{array}$$

where *AE* is the absorbance of the solution when the sample extract is added at a particular level, and *A* is the absorbance of the DPPH solution. The extract concentration providing 50% inhibition (IC50) was calculated from the graph of scavenging effect percentage against extract concentration in the solution.

### 2.7. Sterol Composition

The unsaponifiable fraction was extracted from PSO with diethyl ether, dried, and dissolved in chloroform as described by Lukić et al. (2013) [21]. Identification and quantification of sterols were carried out by capillary gas chromatography on a Varian 3350 GC (Varian Inc., Harbour City, USA) equipped with a VF-5 ms capillary column (30 m × 0.25 mm × 0.25 *μ*m) and FID. Injector, oven, and detector temperatures were 280, 260, and 290°C, respectively, for 40 min. One *μ*l was injected in split mode (1 : 50). Helium was used as a carrier gas with a flow rate of 1.27 ml/min. Thirteen sterols (cholesterol, brassicasterol, 24-methylene-cholesterol, campesterol, campestanol, stigmasterol, Δ^7^-campesterol, Δ^5,23^-stigmastadienol, clerosterol, *β*-sitosterol, sitostanol, Δ^5^-avenasterol, and Δ^5,24^-stigmastadienol) were identified in oil based on their relative retention times with respect to the internal standard, cholestanol, according to the standardized reference method (EEC, 1991, Annexes V and VI). Relative amounts were expressed as proportions (%) of total sterols.

### 2.8. Lipid Class Separation and Fatty Acid Methyl Ester (FAME) Analysis

Total lipids, glycolipids, and phospholipids from grounded seeds were extracted according to Bligh and Dyer (1959) [22]. For the analysis of glycolipids and phospholipids from seed, the lipids were fractionated on silicic acid columns into neutral lipids, glycolipids, and phospholipids by elution with chloroform, acetone, and methanol, respectively. For total fatty acids, glycolipids fatty acids, and phospholipids fatty acids analysis, the lipid extract was directly transesterified by reaction with 14% boron-trifluoride in methanol at 65°C for 30 minutes, after which it was reextracted using hexane and subjected to gas chromatography (GC) analysis. FAMEs analysis was carried out according to the European Union Commission modified Regulation EEC 2568/91 (13) on a Hewlett-Packard gas chromatograph (Hewlett-Packard, Palo Alto, CA), fitted with a flame ionization detector and a split-splitless injector, set at 270°C. The carrier gas was nitrogen (1 mL/min), and elution was performed with a fused silica Agilent DB23 capillary column (60 m length, 0.32 mm inner diameter, and 0.25 *μ*m film thickness). Conditions were as follows: injector temperature, 270°C; flame ionization detector, 280°C; injector split ratio, 1 : 50; the initial column temperature, 130°C; step 1, 6.5°C/min to 170°C; step 2, 2.8°C/min to 215°C, maintained for 12 min; step 3, 40°C/min to 230°C, maintained for 20 min. FAMEs were identified by comparing their relative and absolute retention times to those of authentic cis-fatty acid (CFA) and TFA standards. The FA composition was reported as a relative percentage of the total peak area using a HP Chemstation integrator [23].

### 2.9. Statistical Analysis

Assays were carried out in triplicate. The results are shown as the mean values with standard deviation.

## 3. Results and Discussion

### 3.1. Quality Indexes

The physicochemical properties of pomegranate oil are presented in Table 1. Free acidity is an important quality factor and has been extensively used as a traditional criterion for classifying olive oil in various commercial grades. The free acidity value of PSO is 1.69, significantly lower than the results found by Dadashi et al. (2013) [9] in Iranian varieties (3.78 to 8.36). This acidity is higher than that found in some edible oil such as linseeds oil and sunflower oil indicating that PSO would need refining to make it suitable for edible purposes and suggest that some hydrolytic reactions occur during the extraction [24].

The oxidative state of oils is determined using the peroxide value and specific extinction at 232 and 270 nm, respectively. The peroxide value PV of oil is a valuable index to determine oil quality. If the peroxide value becomes higher than 9 meqO_2_/kg oil, it indicates oxidative corruption in oil [25]. As seen in Table 1, the amount of PV in studied variety is 3.42 ± 0.68 which represents good extraction and maintenance condition. This result indicates that pomegranate seeds oil can be stored for a long times without deterioration, since oils become rancid when the peroxide value ranges from 20 to 40 meqO_2_/Kg oil [26]. Peroxide value of PSO is significantly lower than that of some seeds oils like linseed oil (11.28 meqO_2_/Kg) and sunflower oil (12.87 meqO_2_/Kg) [27].

The specific extinction coefficients at 232 nm and 270 nm are related, respectively, to the degree of primary and secondary oxidation of the oils and thus directly correlate to the amount of peroxide [24, 28].

The values of *k*_232_ (4.15) and *k*_270_ (3.95) are relatively higher than that found in another plant oils such as soybean oil (2.78 and 0.73) [26], sunflower oil (3.83 and 3.65), and olive oil (2.52 and 0.2) [27]. This result confirms that PSO is much oxidized than these oils.

### 3.2. Pigment Content

As shown in Table 1, results show lower content of chlorophylls (0.02) and *β*-carotene (3.17). These results correlate with yellow color of oil.

The level of pigments, however, depends on the stage of fruit ripeness, the extraction process, and storage conditions. Thus, oils extracted from older fruits may contain more carotene pigment or oils from younger fruits contain more chlorophyll pigment [29]. Our fruits are collected in full maturity, which confirms these results.

### 3.3. Total Phenols and Flavonoids

Phenolic content is primary parameter for vegetables quality evaluation and directly involved in the prevention of oxidation and oil preservation. Seeds oils generally contain polyphenols preventing their oxidation [30]. As shown in Table 1, the amounts of total phenols, O-diphenols, and flavonoids in PSO are 93.42, 30.1, and 59.46, respectively. The content of polyphenols in Tounsi variety is lower than that found by Schubert et al. (1999) [3] (15 mg/100 g) in pomegranate cold pressed seed oil. The content of O-diphenols is lower than that found in comparative study of four Iranian pomegranate varieties where the content of O-diphenols can be attained as 58 mg/g [9]. As reported by different studies, the amount of phenolic compounds in olive oil depends on several factors such as cultivar degree of maturation, climate, oil production, and storage [31, 32].

### 3.4. Antioxidant Activity

The antioxidant activity of the PSO was measured by DPPH test. Table 1 shows that the IC50 is 370 *μ*g/ml. Compared to commercial synthetic antioxidant such as BHT (IC50 = 9.12 *μ*g/ml), we concluded that PSO has strong antioxidant activity. This high antioxidant activity can be attributed to the phenolic compounds mainly to richness in O-diphenols. Phenolic compounds have been reported to be present in all vegetable oils, which is very important for the oxidative stability of the polyunsaturated fatty acids of these oils. In fact, linear relationship exists between the phenol content and the oxidative stability of the extra virgin olive oil [33] and the O-diphenols family could be identified as the main source of the overall antioxidant activity and sensorial proprieties of extra virgin oil [32, 34].

### 3.5. Sterols Composition

Sterols are an important nonacylglycerol constituents of vegetable oil because they relate to the quality of the oil and are widely used to check genuineness while it can be used to determine adulteration of an olive oil, and it has recently been suggested that it may be used to classify virgin olive oils according to their fruit variety.

Table 2 shows the sterols composition in PSO. 11 compounds were postulated for wherein the sterol marker was *β*-sitosterol constituting about 77.94% of the total sterols content. The next major components were Δ^5^-avenasterol (7.45%) and campesterol (6.35%). These are followed by stigmasterol (3.21%). All other sterols are present with amounts lower than 3%. Clinical studies have demonstrated that directly intake of phytosterols as a part of the normal diet, or as a supplement, contributes to the reduction of cholesterol levels and the prevention of many diseases and various type of cancers [32]. Recently, phytosterols have been added to vegetable oils as an example of a successful functional food [35]. Our results are slightly different to that found by previous study [1].

### 3.6. Fatty Acid Profile of Seed Oil and Its Lipid Classes

The fatty acid profile of TL and its lipids class were presented in Table 3. Total lipids extracted with the chloroform/methanol mixture were found to be 12.26%. Comparing our results with those previously found, we noticed that the TL content in Tounsi variety was slightly lower than that found by Mekni et al. (2014) [6] in their comparable study between 3 Tunisian pomegranate cultivars in which they reported 15.57%, similar to that found by Melgarejo and Artés (2000) [36] who reported 6.3–12.2% lipids for sweet Spanish pomegranate and much higher than that reported by Hernandez et al. (2000) [8]. These difference can be explained by several factors such as genetic variability, extraction process, and maturation stage [37].

#### 3.6.1. Total Lipids Profile

Total SFA fraction was represented as 35.17% of total lipids. This result is not consistent with those suggested by Mekni et al. (2014) who they found that Gabsi variety seed oil contains 8.51% of total SFA. Among the different components of this fraction, palmitic acid was the main SFA (22.08%) followed by stearic acid (C18:0) with a percentage of 8.94%. Other SFAs such as arachidic acid (C20:0) and behenic acid (C22:0) were presented but in lesser amount within 0.9–1.25%. However, lauric acid and myristic acid were found in insignificant levels. Our results, concerning the dominance of different SFAs, were in good agreement with previous reports [6, 9, 36].

The mean content of UFA is almost 63.17%, in which 16.73% are monounsaturated fatty acids (MUFA), 29.33% di-UFA, and 12.59% of tri-UFA. Major MUFA was oleic acid (C18:1 w9 cis) which accounted for 10.47%, followed by vaccenic acid (C18:1 w7 cis) and palmitoleic acid (C16:1 cis) with amounts of 2.12% and 1.88%, respectively. trans-form is present in trace and the trans/cis ratio of MUFA was 0.026. Our results do not correspond with those found by Mekni et al. (2014) [6] who reported only 8.03% of MUFA, very less amount of oleic acid 0.68%, important percentage of elaidic acid (C18:1 w9 trans) (3.16%) and high trans/cis ratio of MUFA (1.27) in the same variety. On the contrary, our findings coincide with some researchers about other pomegranate varieties. For example, Fadavi et al. (2006) found high amount of oleic acid (17.4%) in some Iranian pomegranate varieties. Similarly, Dadashi et al. (2013) [9] reported high amount of oleic acid (9.36%) and very less amount of elaidic acid (0.06%).

Polyunsaturated fatty acids (PUFAs) fraction represented 46.44% of total lipids in which there were 32.87% cis-form content, 0.35% trans-form content, and 8.92% conjugated form. PSO is characterized by the presence of different types of omega families. In fact, Omega 6 was found to be the major PUFA and it is characterized by the predominance of linoleic acid (C18:2 w6 (c9, c11)) with a content of 28.86% followed by *γ*-linolenic acid (C18:3 w6) (2.82%), while eicosadienoic acid (C20:2 w6) and dihomo-*γ*-linolenic acid (C20:3 w6) were detected in trace.

The predominance of linoleic acid was confirmed in almost PSO studies but its amount was lower than that found in our study. For example, Mekni et al. (2014) [6] reported a level of 7 to 5% of linoleic acid in three Tunisian pomegranate varieties.

Omega 3 PUFAs were represented by the *α*-linolenic acid (C18:3 w3) accounting for 1.02% and eicosatrienoic acid (C20:3 w3) with 0.11%. Compared to others varieties, the amount of *α*-linolenic acid is the highest. For example, Dadashi et al., 2013, reported a level of 0.1 to 0.4%. Others omega families were found like omega 9 and omega 7 and omega 8 but with very few amounts.

Conjugated fatty acids consisted about 13.30% and they represented by three conjugated linoleic acids (CLA) and five conjugated linolenic acids (CLnA) identified by chromatographic mass-spectrometry analysis as different geometric isomers of conjugated linolenic acid and, namely, punicic acid (C18:3 (c9, t11, c13)) is the major isomer (ca. 5.12%), followed by catalpic acid (C18:3 (t9, t11, c13)) (ca. 3.04%), a-eleostearic acid (C18:3 (c9, t11, t13)) (ca. 2.97%), jacaric acid (C18:3 (c8, t10, c12)) (ca. 1.41%), and b-eleostearic (C18:3 (t9, t11, t13)) (ca. 0.45%). Kaufman and Wiesman (2007) [1] reported also four separated peaks of linolenic acid without naming each isomer. According to the literature, PSO is rich in punicic acid and its amount was the highest among all FAs. It ranges from 12.45% [38] to 73.31% [9]. So compared to previous studies, our finding concerning punicic acid amount was the lowest. Conjugated FAs like CLnA are known to have favorable physiological effects such as antitumor activity and body fat reduction [39, 40]. In fact, punicic acid is considered to be an anticancer agent, as demonstrated by its inhibition of human prostate cancer cell invasion [41].

In our study, the MUFA/PUFA ratio was 0.36% which indicates the PUFA richness of PSO so the health-benefiting potential.

#### 3.6.2. Lipids Class and Its Fatty Acids Composition

**Figure 1:**
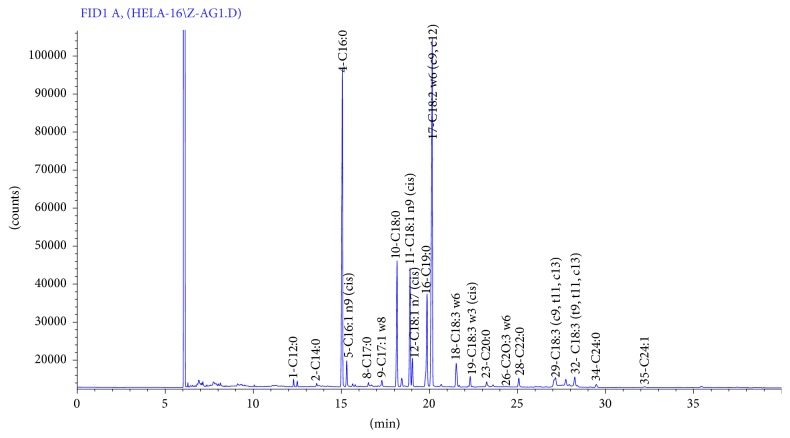
Typical chromatogram of fatty acids analyzed by GC in pomegranate seeds oil.

The total lipids isolated from PSO were fractioned into neutral lipids and polar lipids (namely, also bound lipids) which represented by glycolipids GL and phospholipids PL. Then PL and GL fractions were taken for fatty acid composition study. The results were illustrated in Table 3. Typical chromatogram of studied samples was illustrated in Figure 1. Compared to total lipids, we found that polar lipids were richer in SFA. The amounts of SFA in PL and GL were, respectively, 71.97% and 66.29% wherein palmitic acid and stearic acid were the major SFAs which together comprised more than 91% of total identified SFA. So, it concludes that SFAs were more bound in nature and it might be complexed chemically or physically with carbohydrates or proteins. Besides, it was reported that the ability to stabilize lipids was also affected by the chain length and degree of UFAs. In fact, PL with longer chain length and PL containing more SFA were the most effective antioxidants [42]. Others SFAs like C12:0, C22:0, and C20:0 were detected but in lesser contents. For example, GL contained more amount of C12:0 and C22:0, while PL were richer in C20:0. The ratio of USFA to SFA was lower in polar lipids than in total lipids. Its values were 1.82, 0.39, and 0.45, respectively, in TL, PL, and GL. It was reported that a high ratio of USFA/SFA is regarded favorable for the reduction of serum CT and atherosclerosis and prevention of heart diseases [43].

USFA amounts in GL and PL did not differ significantly from each other. In fact, GL resemble PL in their contents of MUFA in which oleic acid (C18:1 w9 (cis)) was the major MUFA accounting for 8.88% and 7.74%, respectively, in PL and GL fractions.

Concerning PUFA, the amount is slightly higher in GL than in PL. Linoleic acid, the main PUFA, was found to be in similar amount in the two lipid classes, similar to linolenic acid, the next major PUFA. Compared to TL, the ratio of MUFA to PUFA was much higher in polar lipids. The corresponding values for this ratio in PL and GL were, respectively, 0.73% and 0.57%. This indicates that MUFA were more bound in PL than in GL. Conjugated PUFA amount in GL were found to be twofold of that in PL and they were represented mainly by punicic acid and *β*-eleostearic acid.

## 4. Conclusion

In conclusion of this investigation, it is clear that pomegranate seeds give a considerable yield of oil and the oil seems to be a good source of essential fatty acids, phenolics compounds, and phytosterols. Furthermore, the high percentage of PUFA, sterols, and the considerable amount of phenols make it desirable in terms of nutrition and new nonconventional supply for edible purposes and pharmaceutical industries. This work could also serve for developing quality characteristics of PSO.

## Figures and Tables

**Table 1:** Quality, antiradical activity and phenols, flavonoids, and pigments contents in pomegranate seeds oil of Tounsi variety.

	Concentrations
Total phenols (mg/kg)	93.42 ± 1.57
Flavonoids (mg/kg)	59.46 ± 2.68
O-Diphenols (mg/kg)	30.1 ± 5.89
Β-Carotene (mg/kg)	3.17 ± 0.21
Chlorophylls (mg/kg)	0.02 ± 0.0003
Free acidity (%)	1.69 ± 0.00
Peroxide value (meqO_2_/kg)	3.42 ± 0.68
*K*_232_	4.15 ± 0.05
*K*_270_	3.95 ± 0.08
DPPH activity (IC_50_ (mg/ml))	0.37 ± 0.09

Each value represents the mean of three determinations (*n* = 3) ± standard deviation.

**Table 2:** Sterols and triterpenic dialcohols composition (%) of pomegranate oil seeds of Tounsi variety.

	Percent
Cholesterol	0,23 ± 0.02
24-Methylene-cholesterol	0,01 ± 0.00
Campesterol	6,35 ± 0.11
Campestanol	0,08 ± 0.01
Stigmasterol	3,21 ± 0.03
Δ^5,23^-Stigmastadienol	0,05 ± 0.01
Clerosterol	1,23 ± 0.04
*β*-Sitosterol	77,94 ± 0.12
Sitostanol	0,44 ± 0.03
Δ^5^-Avenasterol	7,45 ± 0.07
Δ^5,24^-Stigmastadienol	0,93 ± 0.02
Δ^7^-Stigmastenol	0,27 ± 0.02
Δ^7^-Avenasterol	0,76 ± 0.01
Erythrodiol	0,34 ± 0.020
Uvaol	0,77 ± 0.05

Each value represents the mean of three determinations (*n* = 3) ± standard deviation.

**Table 3:** Fatty acid composition of individual lipid classes of pomegranate seeds.

	Total lipids (%)	Phospholipids (%)	Glycolipids (%)
*C12:0*	0,51 ± 0,05	0,97 ± 0,48	1,19 ± 0,09
*C14:0*	0,36 ± 0,07	0,25 ± 0,06	0,42 ± 0,15
*C14:1*	0,14 ± 0,03	0,05 ± 0,01	0,32 ± 0,02
*C16:0*	22,08 ± 2,71	43,00 ± 1,12	38,25 ± 3,63
*C16:1 w9 (cis)*	1,88 ± 0,28	0,25 ± 0,09	0,30 ± 0,02
*C16:1 w7 (trans)*	0,40 ± 0,01	0,31 ± 0,03	0,37 ± 0,06
*C16:1 w7 (cis)*	0,27 ± 0,07	0,30 ± 0,08	0,45 ± 0,05
*C17:0*	0,54 ± 0,08	0,69 ± 0,02	0,96 ± 0,08
*C17:1 w8*	0,83 ± 0,07	0,53 ± 0,01	0,28 ± 0,02
*C18:0*	8,94 ± 1,41	24,24 ± 1,20	22,40 ± 1,64
*C18:1 w9 (cis)*	10,47 ± 0,76	8,88 ± 0,92	7,74 ± 1,13
*C18:1 w7 (cis)*	2,12 ± 0,23	1,36 ± 0,38	1,13 ± 0,08
*C18:1 w9 (trans)*	0,04 ± 0,02	0,03 ± 0,02	0,13 ± 0,06
*C18:2 (c9, t12)*	0,03 ± 0,00	0,01 ± 0,00	0,13 ± 0,01
*C18:2 (t9, c12)*	0,01 ± 0,00	0,01 ± 0,00	0,14 ± 0,01
*C18:2 w6 (c9, c12)*	28,86 ± 0,26	9,98 ± 0,60	9,60 ± 0,55
*C18:3 w6*	2,82 ± 0,04	3,76 ± 0,88	3,64 ± 1,05
*C18:3 w3 (cis)*	1,02 ± 0,26	0,35 ± 0,08	0,46 ± 0,05
*C18:2 (t9, t11)*	0,10 ± 0,02	0,21 ± 0,08	0,15 ± 0,01
*C18:2 (c11, t13)*	0,10 ± 0,00	0,02 ± 0,01	0,05 ± 0,03
*C18:2 (t10, c12)*	0,16 ± 0,10	0,01 ± 0,01	0,12 ± 0,02
*C20:0*	0,91 ± 0,24	1,41 ± 0,09	1,28 ± 0,12
*C2O:1 w9*	0,43 ± 0,01	0,22 ± 0,20	0,06 ± 0,03
*C2O:2*	0,08 ± 0,00	0,01 ± 0,00	0,08 ± 0,02
*C2O:3 w6*	0,14 ± 0,03	0,01 ± 0,00	0,05 ± 0,00
*C2O:3 w3*	0,11 ± 0,05	0,01 ± 0,01	0,13 ± 0,01
*C22:0*	1,25 ± 0,24	0,90 ± 0,08	1,35 ± 0,26
*C18:3 (c9, t11, c13)*	5,12 ± 0,25	0,68 ± 0,06	1,42 ± 0,10
*C18:3 (c8, t10, c12)*	1,41 ± 0,86	0,03 ± 0,01	0,91 ± 0,24
*C18:3 (c9, t11, t13)*	2,97 ± 2,10	0,03 ± 0,00	0,43 ± 0,07
*C18:3 (t9, t11, c13)*	3,04 ± 1,49	1,20 ± 0,11	1,47 ± 0,37
*C18:3 (t9, t11, t13)*	0,45 ± 0,22	0,05 ± 0,03	0,25 ± 0,04
*C24:0*	0,58 ± 0,19	0,51 ± 0,09	0,44 ± 0,03
*C24:1*	0,15 ± 0,00	0,03 ± 0,04	0,14 ± 0,10
*∑SFA*	35,17 ± 3,74	71,97 ± 2,46	66,29 ± 5,22
*∑MUFA*	16,73 ± 0,20	11,97 ± 1,32	10,91 ± 1,22
*∑PUFA*	46,44 ± 4,57	16,36 ± 1,41	19,02 ± 0,98
*∑cis UFA*	49,33 ± 0,34	25,75 ± 2,64	24,38 ± 2,63
*∑trans UFA*	0,99 ± 0,22	0,60 ± 0,09	0,89 ± 0,16
*conjugated FA*	13,30 ± 4,46	2,03 ± 0,12	4,91 ± 0,73

Each value represents the mean of three determinations (*n* = 3) ± standard deviation.
